# Utilizing Dynamic Phosphorous-31 Magnetic Resonance Spectroscopy for the Early Detection of Acute Compartment Syndrome: A Pilot Study on Rats

**DOI:** 10.3390/diagnostics11040586

**Published:** 2021-03-24

**Authors:** Hiroki Ohta, Nhat-Minh Van Vo, Junichi Hata, Koshiro Terawaki, Takako Shirakawa, Hirotaka James Okano

**Affiliations:** 1 Division of Regenerative Medicine, Research Center for Medical Sciences, The Jikei University School of Medicine, Tokyo 105-8461, Japan; hiro-o@jikei.ac.jp (H.O.); minhvo89@gmail.com (N.-M.V.V.); J.Hata@jikei.ac.jp (J.H.); rtv5v0312@gmail.com (K.T.); 2 Department of Radiological Sciences, Tokyo Metropolitan University, Tokyo 116-0012, Japan; t-shirakawa@tmu.ac.jp

**Keywords:** limb salvage, non-invasive diagnosis method, creatine phosphokinase (CPK), pH, inorganic phosphate (Pi), phosphocreatine (PCr), adenosine triphosphate (ATP)

## Abstract

Introduction: Disasters, including terrorism and earthquakes, are significant threats to people and may lead to many people requiring rescue. The longer the rescue takes, the higher the chances of an individual contracting acute compartment syndrome (ACS). ACS is fatal if diagnosed too late, and early diagnosis and treatment are essential. Objective: To assess the ability of dynamic phosphorus magnetic resonance spectroscopy (^31^P-MRS) in the early detection of muscular damage in ACS. Materials and Methods: Six ACS model rats were used for serial ^31^P-MRS scanning (9.4 Tesla). Skeletal muscle metabolism, represented by the levels of phosphocreatine (PCr), inorganic phosphate (Pi), and adenosine triphosphate (ATP), was assessed. The PCr/(Pi + PCr) ratio, which decreases with ischemia, was compared with simultaneously sampled plasma creatine phosphokinase (CPK), a muscle damage marker. Results: The PCr/(Pi + PCr) ratio significantly decreased after inducing ischemia (from 0.86 ± 0.10 to 0.18 ± 0.06; *p* < 0.05), while CPK did not change significantly (from 89 ± 29.46 to 241.50 ± 113.28; *p* > 0.05). The intracellular and arterial pH index decreased over time, revealing significant differences at 120 min post-ischemia (from 7.09 ± 0.01 to 6.43 ± 0.13, and from 7.47 ± 0.03 to 7.39 ± 0.04, respectively). In the reperfusion state, the spectra and pH did not return to the original values. Conclusions: The dynamic ^31^P-MRS technique can rapidly detect changes in muscle bioenergetics. This technique is a promising non-invasive method for determining early muscular damage in ACS.

## 1. Introduction

Acute compartment syndrome (ACS) of the extremities, a syndrome that frequently occurs in disaster-prone countries such as Japan, can cause permanent damage to the muscles, nerves, and vasculatures [1,2,3]. Moreover, if left untreated for more than 8 h, limb salvage may be impossible, and it may even cause death [4,5,6]. Thus, early detection and monitoring of the disease progression are essential for evaluating skeletal muscle status, and differentiating reversible and irreversible tissue damage in skeletal muscles.

The initial suspicion of ACS is mostly inferred from observing its classical features, such as pain, pallor, pulselessness, paresthesia, poikilothermy, and paralysis [7]. It is verified either through invasive or non-invasive methods [8,9,10]. A typical invasive method is through measuring and calculating the difference between the mean arterial pressure and the intra-compartmental pressure (ICP) [11]. An absolute ICP of 30 mmHg indicates the immediate need for a fasciotomy. Another invasive procedure is the use of a serum biomarker, specifically creatine phosphokinase (CPK) [12]. A CPK concentration of greater than 4000 U/L is associated with ACS in humans [13]. However, these invasive procedures are not sensitive in the early detection of ACS [7,14,15].

Current non-invasive ACS detection techniques are near-infrared spectroscopy (NIRS), ultrasound, laser Doppler flowmetry, and conventional magnetic resonance imaging (MRI). However, as these are used to monitor changes occurring to the body structures in real-time, they are also incapable of detecting early signs of ACS [16]. Thus, early ACS detection remains a difficult task, as conventional diagnostic techniques often provide insufficient evidence to diagnose a patient’s condition [16].

Selecting the most appropriate diagnostic technique requires a good understanding of ACS’s pathophysiology [17]. For the early diagnosis and treatment of ACS, it is necessary to evaluate muscle bioenergetics and metabolism non-invasively [7,16,18,19,20,21,22]. Magnetic resonance spectroscopy (MRS) is a unique technique for assessing tissue metabolic properties through the quantification of essential metabolites such as inorganic phosphate (Pi), phosphocreatine (PCr), and adenosine triphosphate (ATP) [22,23]. Phosphorus magnetic resonance spectroscopy (^31^P-MRS) has been used in clinical practice to evaluate high-energy phosphate metabolism changes of muscular and peripheral arterial diseases in humans [21,24,25]. ^31^P-MRS can dynamically monitor the rate of high-energy phosphate depletion and resynthesis [26]. Additionally, it can measure the intracellular pH and mitochondrial oxidative capacity [19,27]. These are valuable indices that can be used to differentiate between normal and pathological states.

In clinical practice, most MRI machines used to detect ACS have a magnetic field strength of under three Tesla. However, with the development of hardware technology, it is becoming possible to maintain the uniformity of magnetic field at a strength higher than three Tesla [28,29,30,31,32]. Therefore, the clinical utility of ultra-high magnetic field systems is being explored [33,34]. In our pilot experiment, we used a ^31^P-MRS with an ultra-high magnetic field (9.4 Tesla) to gain a higher signal-to-noise ratio (SNR), shorten the data acquisition time, improve spectra quality, and reduce metabolite overlapping. Doing so made the quantification of energy metabolism more accurate [35,36,37].

We hypothesize that ^31^P-MRS can provide information on bioenergetic changes in ACS. This study aimed to examine whether dynamic ^31^P-MRS can detect early skeletal muscle bioenergetic changes in ACS. We compared results from ^31^P-MRS with blood tests to offer essential information about muscle pathophysiology in the pre-ischemic, ischemic, and reperfusion phases.

## 2. Materials and Methods

### 2.1. Animals

Male normotensive Sprague-Dawley rats (*n* = 6; 9 to 12 weeks old; 276–499 g), obtained from Nihon SLC (Japan SLC, Inc., Shizuoka, Japan), were housed in individual cages in a controlled room (temperature: 24–25 °C; humidity: 50–60%) with a 12:12 hour light-dark cycle. The rats were allowed free access to food (CE-2, CLEA Japan, Inc., Tokyo, Japan) and water [38]. The sample size of this pilot study was determined by the research team’s shared experience, personal judgment of the principal investigators, and in light of previous animal studies [35,39,40,41,42].

The research protocol was approved by the Institutional Animal Care and Use Committee of the Jikei University School of Medicine (protocol number: 2019-043C1). All experimental procedures were conducted under the Fundamental Guidelines for Proper Conduct of Animal Experiments and Related Activities in Academic Research Institutions, issued by the Japanese Ministry of Education, Culture, Sports, Science and Technology [38]. Such conduct includes upholding ethical values of non-maleficence, among others.

### 2.2. Creation of the Fastened Zip-Tie Rat Model

**Figure 1:**
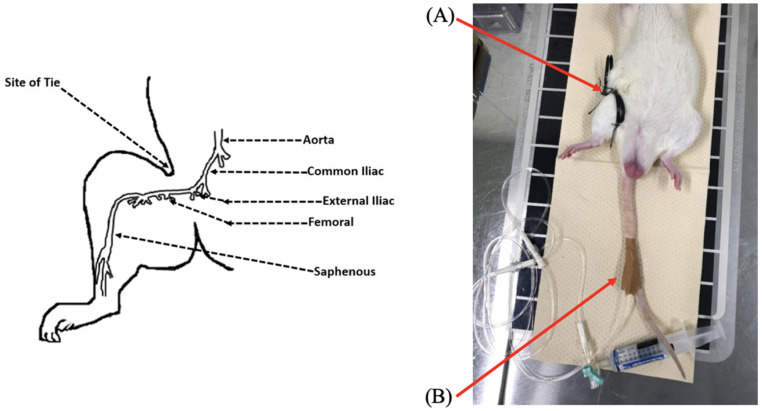
Fastened zip-tie rat model of acute compartment syndrome. Creation of an ACS model of the entire right lower limb by wrapping a zip tie around the rat’s right inguinal region. (**A**) The site of the zip tie. (**B**) The arterial line for blood sample collection. An arterial line was placed in the caudal ventral artery and set up so that blood could be sampled at any time.

A vascular surgeon performed the fastened zip-tie rat model of ACS. Anesthesia was induced using 3% isoflurane kept within the laboratory “Small Animal Anesthesia System” (SBN-487, Shinano, Tokyo, Japan), and titrated to maintain an acceptable standard during the experiment. This isoflurane concentration is commonly used for magnetic resonance (MR) procedures as it does not impact the results obtained [43,44]. Respiratory functioning was monitored in real-time using an MR-compatible small animal monitoring and gating system (SA Instruments, Inc., Stony Brook, NY, USA). Once the anesthesia had taken effect, the rats were placed on a heating pad in a supine position. Then, two plastic zip ties (ELPA, Osaka, Japan; length: 150 mm, width: 3.6 mm) were loosely attached to the intended area to induce ischemia, and were sewn at three points (two points supine, one point on the lateral side) to the rat’s inguinal region while avoiding vascular damage (Figure 1). Then, pre-ischemic scanning was undertaken, followed by the placement of MR coils on the hindlimb’s lateral side and tightening of the zip ties to induce ischemia.

**Figure 2:**
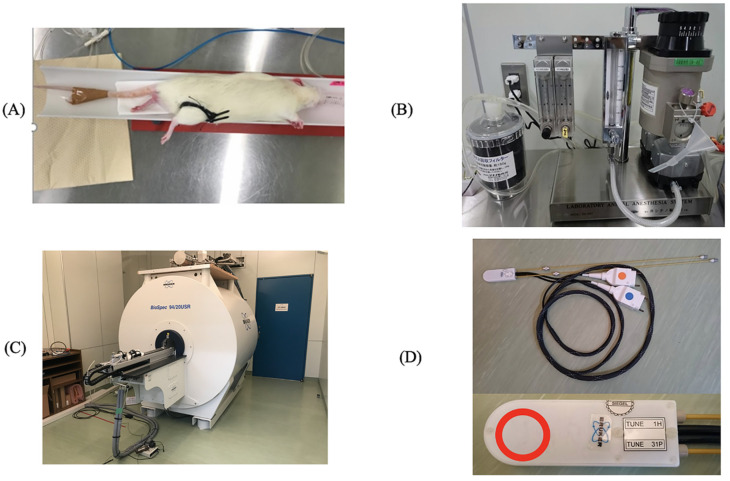
The experiment’s equipment. The prepared rat was placed in a left lateral position (**A**) on a dedicated MR cradle and connected to an anesthesia system (**B**), where the inhalation concentration could be adjusted accordingly. An ultra-high field 9.4 Tesla magnetic resonance imaging (MRI) machine (**C**) and a surface coil capable of observing the biochemical kinetics of ^1^H and ^31^P (**D**) were fixed to the right lower leg region. The red circle (20 mm inner diameter) is the region of interest.

After preparation of the fastened zip-tie rat model of ACS, the rats were placed in a supine position and immobilized on the MR cradle with the hindlimb in full extension. The MR cradle was connected to a small animal ventilator (SA Instruments, Inc., Stony Brook, NY, USA). The rat’s respiration rate was continuously monitored (SA Instruments, Stony Brook, NY, USA) and controlled at 80–120/minute throughout the MR scanning. Body temperature was maintained at about 37 degrees Celsius by heating bath circulator equipment (CW-05G, Lab Companion, Daejeon, Korea) connected to the MR cradle. All equipment used is shown in Figure 2. After 120 min of ischemia, the zip ties were cut to resume perfusion.

### 2.3. Experimental Procedure

**Figure 3:**
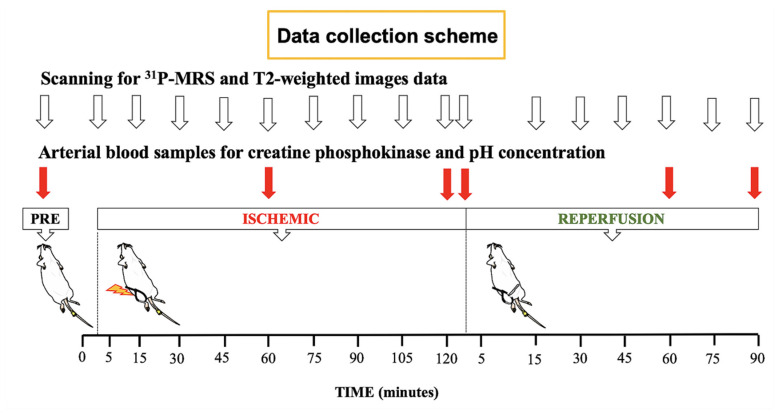
The experimental protocol. White arrows indicate the timings of data collection for phosphorus magnetic resonance spectroscopy (^31^P-MRS) and T2-weighted images. Red arrows designate the time of arterial blood sampling for creatine phosphokinase and pH concentration. ^31^P-MRS spectra were acquired at rest, during muscle compression (for 120 min) and reperfusion (for 90 min). During the magnetic resonance (MR) procedure, the concentration of pH and creatine phosphokinase (CPK) were analyzed from blood samples obtained from the tail artery.

We acquired ^31^P-MRS spectra, hydrogen MRI images (^1^H-MRI), and blood samples from rats induced with ACS through the use of zip ties. Pre-ischemic and ischemic states were monitored continuously from time zero to 120 min. After releasing the zip ties, the reperfusion state was followed for 90 min [26]. ^31^P-MRS spectra and blood samples were acquired at rest, during muscle compression (120 min), and after inducing reperfusion (90 min) (Figure 3). During the MR procedure, the concentration of pH and CPK were analyzed by blood samples obtained from the tail artery. The rats were able to walk normally at 2 days follow-up.

### 2.4. Assessing ^1^H-MRI and ^31^P-MRS

Measurements were performed on an MR scanner, Biospec, 9.4 Tesla (Bruker Optik GmbH, Ettlingen, Germany) and controlled using the Paravision 6.2 software package (Bruker Biospin). The rat’s knee and leg were fully extended, with the knee centered over the surface coil, and Bruker dual resonance linearly polarized coils ^1^H/^31^P (^1^H: 400.525 MHz and ^31^P: 162.056 MHz) with 20 mm inner diameter, attached underneath the hind limb to perform shimming. The leg was secured to the ^1^H/^31^P dual coil covered with masking tape to avoid motion during the experiment.

Localizer ^1^H-images were acquired to detect the leg’s position within the sensitive area of the ^1^H/^31^P dual coil. Subsequently, wobble adjustment was performed that allowed for the radiofrequency (RF) coil’s (measuring its absorption spectra) manual tuning/matching. We selected two coils (elements ^1^H and ^31^P, respectively) with an isotropic voxel of 48 × 48 × 48 mm^3^ in the surface coil’s sensitive field. The wobble curve was tuned and matched until the dip reached the center, and its minimum was close to zero. After that, the external magnetic field (B_0_) map, used to measure a field map of the object used in the study to calculate shims, was set up. The shim was calculated based on a previously measured B_0_-field map to optimize the field homogeneity within the shim volume. As a result, a proton linewidth of 120–140 Hz was obtained. When the line width at half the height of the proton signal was about 0.5 ppm for one free induction decay (FID), the magnetic field homogeneity was accepted. Then, the spectrometer was turned to ^31^P nuclei. The entire procedure took about five minutes on average.

**Figure 4:**
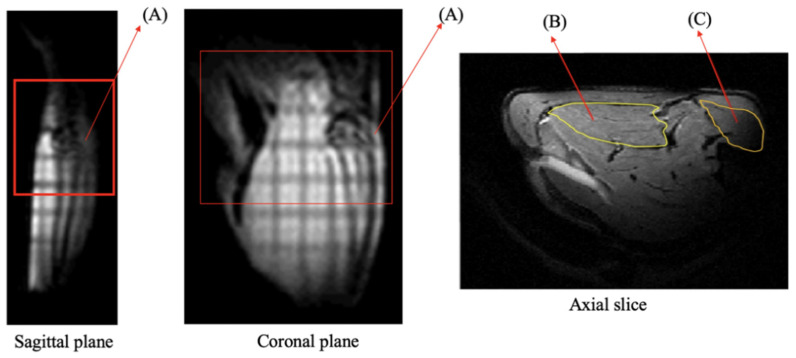
T2-weighted image scanning plan for the detection of the compartment region in the rats. The sagittal and coronal planes are the localizer views. A: Shows the knee joint. The red box indicates the range of the scanner’s field of view. The axial slice was localized from the sagittal and coronal planes. B: The locations correspond to the gastrocnemius muscle (GA), which is located at the posterior side of the leg. C: Tibialis anterior muscle (TA), which is located on the anterior side of the leg. The yellow mark indicates the border of the muscles.

^31^P spectra were acquired, followed by T2-weighted images, leading to a total acquisition time of 10 min during the pre-ischemic phase. The ^31^P-MRS dynamic protocol consisted of 15 min for the pre-ischemic phase, 120 min for the ischemic phase, and 90 min for the recovery phase, respectively, to quantify phosphate metabolite changes. Each acquisition of ^31^P-MRS (using non-localized spectra with a single pulse technique) was acquired with the following parameters: flip angle = 90°; repetition time (TR) = 2000 ms; average = 192. Total acquisition time was 6 min 24 s. The parameters used for axial slice T2-weighted images (Figure 4) were as follows: “fast spin-echo sequence”; TR = 2000 ms; time to echo (TE) = 30.69 ms; refocusing angle = 143.7°; rare factor = 8; averages = 3, matrix = 256 × 256 pixels; field of view = 35 × 50 mm^2^; slice thickness = 1.00 mm, slices = 18; scanning time = 3 min 12 s). The experimental procedure workflow is illustrated in Figure 3.

The MRS data were first processed with TopSpin 4.0.7 software (Bruker Biospin Corp., Billerica MA). The resulting datasets were fitted in the time domain using the AMARES (advanced method for accurate, robust, and efficient spectral fitting) algorithm, implemented in jMRUI software. The NMRSCOPE tool (jMRUI software package) created a basic set of five metabolite spectra that included Pi, PCr, and ATP. PCr and Pi peaks were fitted to Lorentzian line shapes, whereas µ-ATP, α-ATP, and β-ATP signals were fitted to Gaussian line shapes [45]. PCr was used as an internal reference for calculating the absolute concentration of Pi and ATP [46,47]. The PCr/(Pi + PCr) ratio, a marker of the energy state level, was calculated from the Pi and PCr areas [27]. The intracellular pH was calculated from the chemical shift of Pi relative to PCr, utilizing the following equation:

pH = 6.75 + log (δ − 3.27/(5.69 − δ),(1)

where δ is the chemical shift of the Pi peak in parts per million relative to PCr [21].

T2-weighted images were analyzed using ImageJ software (Rasband, W.S., ImageJ, U. S. National Institute of Health, Bethesda, MD, USA) [48]. The signal intensity was measured in two compartments corresponding to the tibialis anterior and the gastrocnemius muscles (locations indicated in Figure 4). The signal intensities of these two regions of interest (ROI) were calculated as the mean values of all pixels within the ROI. The tibia bone’s signal intensity was then used as the reference value to normalize the signal intensity. The signal intensity through each phase, from the pre-ischemic phase to the recovery phase, was quantified.

### 2.5. Examining Blood Samples

Blood samples were collected for ex vivo analysis. A 24-gauge catheter for invasive blood sampling collection was inserted into the ventral tail artery. After sampling arterial blood in the pre-ischemic state, heparinized physiological saline was flushed into the catheter. The catheter was kept in place until the end of the experiment. Each blood sample (0.25 mL) was obtained and placed into the i-STAT CG4 + cartridge (Abbott product, USA) and FUJI DRI-CHEM slides (Fujifilm Medical, Tokyo, Japan) of the blood test kit. Blood samples were obtained in the pre-ischemic and ischemic states (60 min and 120 min), and after pressure removal (5 min, 60 min, and 90 min). It was assessed at each time point for measurement of the inflammatory biomarker CPK.

Blood samples were analyzed using an automatic biochemical analyzer (FUJI DRI-CHEM 3500v, Fujifilm Medical, Tokyo, Japan) and handheld blood analyzer i-STAT 1 (Abbott product, Princeton, NJ, USA). Results were obtained after 2 min. The analysis included pH and CPK [13,49]. Because the blood analyzer cannot display values over 2000 U/L, we decided to declare results that exceeded the upper boundary as 2000 U/L.

### 2.6. Statistical Analysis

The results are presented as the mean ± SD. A repeated-measures nonparametric Friedman test was used to compare ^31^P-MRS and CPK between time points. Potential relationships between the relative signal intensity of the T2-weighted images and CPK were assessed using nonparametric Spearman rank-order correlation. Nonparametric tests were done in adherence with conventional statistical guidelines, as the sample size was small [50]. Statistical significance was *p* < 0.05. Statistical analyses were performed using GraphPad Prism (version 8.0.2, GraphPad Software, Inc., SanDiego, CA, USA). Significant differences are indicated by *p* values in the figures.

## 3. Results

**Figure 5:**
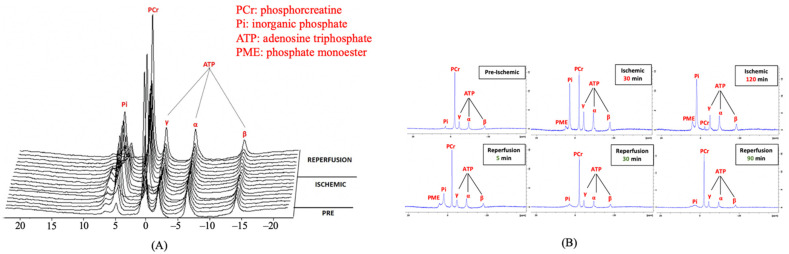
The dynamic graph of the ^31^P-MRS spectrum. (**A**) Time series of the dynamic ^31^P-MRS spectrum in the pre-ischemic, ischemic, and reperfusion states. (**B**) The change of the ^31^P-MRS wave during the experiment. In the ischemic state, a phosphate monoester (PME) peak appeared at 30 min and gradually increased until 120 min of the ischemic state. At the beginning of the reperfusion state, the PME peak started to decrease, and completely disappeared at 185 min of the reperfusion state.

**Figure 6:**
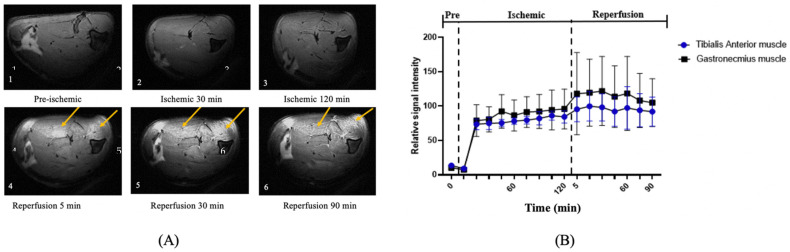
The change of relative signal intensity of T2-weighted images during ischemic and reperfusion states. (**A**) T2-weighted images during the experiment. Images at (1) pre-ischemia, (2) 60 min of ischemia, (3) 120 min of ischemia, and (4) 5 min, (5) 60 min, and (6) 90 min of reperfusion. Hyperintensity was seen at the beginning of the reperfusion phase. (**B**) The mean signal intensity in T2-weighted images. The mean signal intensities were calculated at two regions during the experiment (GA and TA muscles). The increasing relative signal intensity of T2-weighted images showed that the water content increased in the extracellular space. It demonstrated that the edema phenomenon gradually appeared in the reperfusion phase.

^1^P-MRS: Upon inducing ACS, ^31^P-MRS detected changes in the muscles at all time points, but assessing derived parameters such as spectra helped identify muscle changes, as shown in Figure 5. The metabolite concentration of PCr, Pi, and ATP was documented as baseline values. Typical T2-weighted fast spin-echo images are shown in Figure 6.

**Figure 7:**
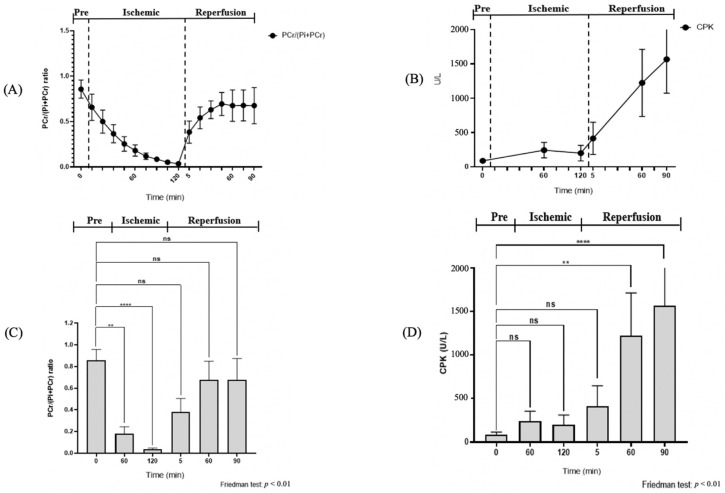
Dynamics in the ratio of PCr/(Pi + PCr) and concentration of CPK during the ischemic and reperfusion states. (**A**,**B**) Changing of the PCr/(Pi + PCr) ratio and CPK of rat skeletal muscles during 2 h of the ischemic and 1.5 h of the post-ischemic states. (**C**,**D**) Comparison of the quantitative PCr/(Pi + PCr) ratio and CPK at 0, 60, 120, 125, 185, and 215 min before, during, and after ischemia. ns (not significant); ** (0.001 < *p* < 0.005) and **** (*p* < 0.0001), based on the Friedman test.

After muscle compression, the PCr/(Pi + PCr) ratio displayed a steep fall of 0.68 from the pre-ischemic value, and continued to decline for up to 120 min of ischemia (Table 1). The Friedman test revealed a significant difference in the PCr/(Pi + PCr) ratio between the pre-ischemic state, 60 min of ischemia, and 120 min of the ischemic state (*p* < 0.05, Figure 6). Additionally, there was a significant change of intracellular pH after 120 min of ischemia (*p* < 0.05, Figure 7, Table 2). At 30 min of the ischemic state, a phosphate monoester (PME) peak was detected, which was not seen in the pre-ischemic phase, in the region of about 4.7–5.0 ppm to the left of the Pi peak (Figure 5). The signal intensity of the tibialis anterior (TA) and gastrocnemius (GA) muscles gradually increased in the T2-weighted images.

Once the zip ties were released, the ^31^P-MRS spectra displayed instantaneous gradual recovery to the pre-ischemic values. Nevertheless, the PCr/(Pi + PCr) ratio did not ultimately return to normal values, with a difference of 0.19 at 90 min of the reperfusion state (Table 1). The intracellular pH also increased during the reperfusion time, but did not recover to its baseline values (Table 2). The PME peak gradually decreased and disappeared during the reperfusion state (Figure 5).

After release, the muscles’ signal intensities became inhomogeneous, wherein they were remarkably higher than those of other areas (Figure 6). At 90 min of the reperfusion state, the high signal intensity of both the TA and GA muscles was preserved. The signal intensity of the two regions was obtained as relative values to the mean baseline signal (Table 3).

Blood samples: No significant changes were seen in the CPK concentration during the ischemic state when compared to the pre-ischemic level (*p* > 0.05, Figure 7); only slight changes were observed (86 ± 29.47). CPK gradually increased throughout the experiment period. At 90 min of reperfusion, the CPK increased more rapidly (1566.17 ± 493.30 U/L).

**Figure 8:**
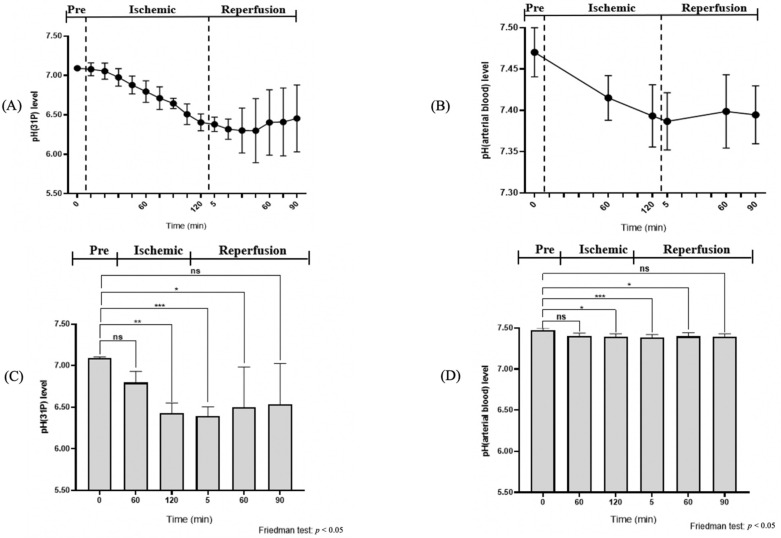
Dynamics in the concentration of intracellular pH and systemic pH during the ischemic and reperfusion states. (**A**,**B**) The change of the intracellular pH and arterial blood pH value during the experiment. (**C**,**D**) Comparison of the quantitative intracellular pH and arterial pH in pre-ischemia, ischemia, and reperfusion. ns (not significant); * (0.01 < *p* < 0.05), ** (0.001 < *p* < 0.005) and *** (*p* < 0.001), based on the Friedman test.

The arterial blood pH level also revealed a minuscule change from pre-ischemia (7.47 ± 0.03) to 60 min of ischemia (7.42 ± 0.03). However, at 120 min of ischemia, a significant change was documented. At 90 min of reperfusion, it had not fully recovered to its pre-ischemic value (Figure 8, Table 2).

**Figure 9:**
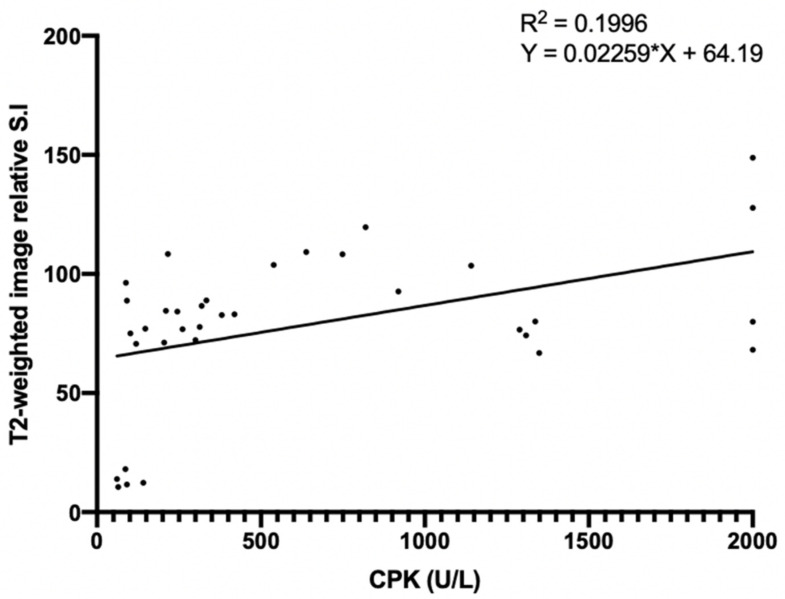
The correlation between the relative signal intensity of T2-weighted images and the CPK concentration. * (0.01 < *p* < 0.05).

There was a significant correlation between the relative signal intensity of the T2-weighted image and CPK concentration from blood examination (R^2^ = 0.1996, *p* < 0.05), revealing the presence of edema at the site of the ACS (Figure 9).

There was a significant correlation of the relative signal intensity of T2-weighted images with the CPK concentration, based on blood examination. The gradual increase in the T2 signal intensity and CPK level demonstrated the edema phenomenon.

## 4. Discussion

The most important finding of this study is that ^31^P-MRS can detect the change of muscle bioenergetics that occurs during the early ischemic state in rats. This could potentially be a reliable non-invasive method for the early detection of ACS in humans; more research is warranted. ^31^P-MRS provided a reliable, sensitive measure of the muscle metabolites changes that occur during ACS. These data are consistent with previous data from the arterial occlusion model, wherein ^31^P-MRS was found to provide early detection of muscle bioenergetic changes [40]. ^31^P-MRS spectra displayed real-time responsiveness with an immediate detectable change at a constant level when plastic zip ties were fastened, suggesting a loss of perfusion to the tissues. These data revealed that the ratio of PCr/(Pi + PCr) was firmly lower than the baseline value during the ischemic state. In contrast to this, the CPK concentration only showed a slight change in the ischemic state. Thus, it could be suggested that ^31^P-MRS can detect bioenergetic changes more rapidly and sensitively than CPK. However, CPK can still be used as a useful biomarker to indicate the extent of muscle damage in the sub-acute phase [51]. Moreover, CPK has long been used as a primary biomarker in recognizing trauma for patients with ACS [13]. It is essential to differentiate between invasive measures, such as blood CPK, and non-invasive measures, such as ^31^P-MRS.

During the ischemic and recovery phase, we consistently found that the PME was peaking. This is an additional sign that can potentially help in detecting early muscle damage in ACS [52]. While it is possible to perform ^31^P-MRS at a more clinically common field strength of 3 Tesla, differentiation of peaks in the spectra will be more difficult and less efficient [34]. Nowadays, a magnetic field MRI higher than 3 Tesla with ^31^P-MRS is used to detect the early stage of disease [36,53]. Thus, ^31^P-MRS has been applied more often in clinical practice to diagnose muscle diseases. The results of this study show there is merit in using ^31^P-MRS for peripheral vascular disease detection as well, specifically ACS. The ^31^P-MRS method exhibited potential for use in diverse settings.

Edema of the hindlimb’s compartment, depending on the location in the muscles, was determined by ^1^H-MR images. During the reperfusion phase, the elevated relative signal intensity of T2-weighted images showed that the water content increased in the extracellular space, similar to other studies [54]. Additionally, there is a correlation between the relative signal intensity of the T2-weighted images (TA and GA muscle regions) and CPK. After the release of muscle compression, it is understood that apparent edema around the skeletal muscle occurred due to the increased vascular permeability in the skeletal muscle induced by oxygen-derived free radicals. This result demonstrated that the muscle damage was consistent for edema symptoms, and thereby suggests that alterations in skeletal muscle high-energy phosphate metabolism occur early in the pathophysiology of ACS. Thus, ^31^P-MRS can potentially be a useful non-invasive method for the early diagnosis of ACS, thereby increasing the probability of limb salvage in these patients.

In our study, there was an evident difference between the control pH of ^31^P-MRS and the blood test during the ischemic state. The pH decreased more extensively in ischemic tissue than in the rest of the body. Anaerobic glycolysis lowered the pH in the ischemic area. However, as homeostasis tightly controls the systematic pH value, the value was almost unchanged (normal mammalian blood pH = 7.40 ± 0.02). Hence, this observation supports a previous study which asserted a difference between the arterial blood pH and the superficial pH value [55].

However, the present study has several limitations. First, as we only utilized six rats, the sample size was evidently small, which greatly affected the generalizability of the findings of this study due to a lack of variability. Nonetheless, this pilot study is useful to provide the groundwork for future studies [56]. Second, the surface coil’s sensitivity area was limited. Thus, standardization of images was not possible, rendering the images incomparable. Regardless, it proved the presence of edema in the ACS model [40]. Third, similar to most cases of the reperfusion state, the blood analyzer equipment could not display the specific value of the CPK concentration as it exceeded the upper limit of measurement (2000 U/L). However, it was considered that detailed numerical measurement was unnecessary because it was sufficiently high when compared to the pre-ischemic level. Lastly, after the experiment, we did not conduct a follow-up on the rats’ health status by MRS. We only observed the rats’ behaviors. In the next phase of this study, we recommend conducting a follow-up on the health of the animals post-experiment.

There is merit in comparing the ^31^P-MRS and blood samples of patients with ACS to further enhance the clinical utility of this technique. There is also tremendous research in the field of regenerative medicine, with possible application in emergency medicine. It has been reported that the clinical efficacy of mesenchymal stem cell therapy for ischemic diseases [57]. Therefore, mesenchymal stem cell therapy has the potential for ACS. By advancing this research, we hope to improve the survival of patients rescued from disasters, and to prevent the deterioration of quality of life due to unnecessary amputation of the lower limbs. 

## 5. Conclusions

Our study showed the possibility of using ^31^P-MRS as a non-invasive, reliable, and sensitive assessment of changes in muscle metabolites. Acquiring clinical data via ^31^P-MRS with MRI ultra-field 9.4 Tesla may have the benefit of increased SNR and spectra quality. This research demonstrated that dynamic ^31^P-MRS measurements may be faster and more accurate than blood sampling tests. Dynamic ^31^P-MRS could be useful for real-time detection of early ischemic muscular damage in ACS.

## Figures and Tables

**Table 1:** PCr/(Pi + PCr) ratio and creatine phosphokinase (CPK) level at different time points.

	Pre-Ischemic	Ischemic		Reperfusion		
Minute	0	60	120	5	60	90
PCr/(Pi + PCr)						
Case 1	0.85	0.15	0.02	0.29	0.46	0.46
Case 2	0.91	0.23	0.03	0.28	0.48	0.41
Case 3	0.89	0.24	0.05	0.59	0.80	0.84
Case 4	0.66	0.07	0.05	0.37	0.85	0.87
Case 5	0.90	0.20	0.03	0.30	0.81	0.80
Case 6	0.93	0.19	0.03	0.46	0.67	0.66
	0.86 ± 0.10	0.18 ± 0.06	0.04 ± 0.01	0.38 ± 0.12	0.68 ± 0.17	0.67 ± 0.20
	(0.85–0.91 *)	(0.15–0.23 *)	(0.03–0.05 *)	(0.29–0.46 *)	(0.48–0.81 *)	(0.46–0.84 *)
		*p* = 0.003 **	*p* < 0.0001 **	*p* = 0.067	*p* > 0.999	*p* > 0.999
CPK level						
Case 1	71	147	334	819	2222	2222
Case 2	61	102	245	205	1309	2222
Case 3	65	300	313	381	1289	1337
Case 4	91	419	91	216	749	919
Case 5	87	261	119	539	1349	2222
Case 6	141	220	88	319	638	1141
	89 ± 29.46	241.50 ± 113.28	198.33 ± 112.88	413.16 ± 233.51	1259.33 ± 562.95	1677.16 ± 611.31
	(65–87 *)	(147–220 *)	(91–313 *)	(319–539 *)	(749–1349 *)	(919–2222 *)
		*p* = 0.948 **	*p* > 0.999 **	*p* = 0.126 **	*p* = 0.001 **	*p* < 0.0001 **

Mean ± SD (*n* = 6). The unit of PCr/(Pi + PCr) and creatine phosphokinase level are ratio and U/L, respectively. * Interquartile range *p* value in relation to the pre-ischemic state. ** shows significant difference.

**Table 2:** Intracellular pH (^31^P-MRS) and pH (arterial blood) at different time points.

	Pre-Ischemic	Ischemic		Reperfusion		
Minute	0	60	120	5	60	90
pH (^31^P-MRS)						
Case 1	7.11	6.85	6.47	6.39	6.54	6.53
Case 2	7.08	6.84	6.41	6.35	6.44	6.57
Case 3	7.10	6.85	6.55	6.54	7.02	7.06
Case 4	7.08	6.52	6.20	6.23	6.09	6.13
Case 5	7.08	6.83	6.46	6.37	5.84	5.84
Case 6	7.10	6.88	6.49	6.49	7.05	7.07
	7.09 ± 0.01	6.80 ± 0.14	6.430 ± 0.13	6.40 ± 0.10	6.50 ± 0.49	6.53 ± 0.49
	(7.08–7.10 *)	(6.83–6.85 *)	(6.41–6.49 *)	(6.35–6.49 *)	(6.09–7.02 *)	(6.13–7.06 *)
		*p* = 0.614 **	*p* = 0.004 **	*p* = 0.0008 **	*p* = 0.021 **	*p* = 0.083 **
pH (arterial blood)						
Case 1	7.50	7.38	7.35	7.35	7.36	7.36
Case 2	7.45	7.44	7.40	7.38	7.39	7.39
Case 3	7.47	7.42	7.39	7.38	7.41	7.39
Case 4	7.50	7.43	7.45	7.45	7.48	7.46
Case 5	7.43	7.38	7.35	7.36	7.38	7.38
Case 6	7.46	7.36	7.38	7.37	7.36	7.36
	7.47 ± 0.03	7.41 ± 0.03	7.39 ± 0.04	7.39 ± 0.04	7.40 ± 0.04	7.39 ± 0.034
	(7.45–7.50 *)	(7.38–7.43 *)	(7.35–7.40 *)	(7.36–7.38 *)	(7.47–7.41 *)	(7.36–7.39 *)
		*p* = 0.103 **	*p* = 0.021 **	*p* = 0.0008 **	*p* = 0.043 **	*p* = 0.103 **

Mean ± SD (*n* = 6). * Interquartile range *p* value in relation to the pre-ischemic state. ** shows significant difference.

**Table 3:** The signal intensity values of the anterior tibialis muscle (TA) and gastrocnemius muscle (GA).

	Pre-Ischemic	Ischemic		Reperfusion		
Minute	0	60	120	5	60	90
TA						
Case 1	14.58	76.99	88.92	119.68	148.83	127.75
Case 2	13.88	75.03	84.21	71.18	74.22	68.17
Case 3	10.58	72.20	77.73	82.73	76.57	80.06
Case 4	11.61	83.04	88.80	108.35	108.29	92.60
Case 5	18.08	76.87	70.70	103.82	66.78	79.96
Case 6	12.36	84.52	96.29	86.58	109.18	103.53
	13.52 ± 2.67 (11.61–14.58 *)	78.11 ± 4.75 (75.03–83.04 *)	84.44 ± 9.09 (77.73–88.92 *)	95.39 ± 18.18 (82.73–108.35 *)	97.31 ± 31.02 (74.22–109.18 *)	92.01 ± 21.29 (79.96–103.53 *)
GA						
Case 1	6.08	130.85	153.34	238.48	213.38	151.92
Case 2	11.78	77.82	86.81	88.18	67.87	64.29
Case 3	8.05	87.20	85.93	103.56	135.08	141.16
Case 4	10.99	70.50	94.75	97.95	109.43	92.68
Case 5	16.43	78.55	74.09	103.50	68.74	78.24
Case 6	8.22	74.15	80.41	77.50	116.45	102.29
	10.26 ± 3.67 (8.05–11.78 *)	86.51 ± 22.42 (74.15–78.55 *)	95.89 ± 28.98 (85.93–94.75 *)	118.20 ± 59.78 (88.18–103.56 *)	118.49 ± 53.67 (67.87–116.45 *)	105.10 ± 34.76 (78.24–102.29 *)

Mean ± SD (*n* = 6). * Interquartile range *p* value in relation to the pre-ischemic state.

## Data Availability

Data were not collected from a public database. All relevant data are available upon request from the corresponding author.

## Abbreviations

ACS: acute compartment syndrome; B_0_: external magnetic field; SE: spin echo; SI: signal intensity; TR: time repetition; TE: echo time; MRI: magnetic resonance imaging; ^31^P-MRS: ^31^phosphorus magnetic resonance spectroscopy; TA: tibialis anterior; GA: gastrocnemius; PCr: phosphocreatine; Pi: inorganic phosphate; ATP: adenosine triphosphate; SNR: signal to noise ratio; CK: creatine kinase; CPK: creatine phosphokinase; PME: phospho-monoester.
